# Desflurane Versus Sevoflurane and Postoperative Cardiac Biomarkers in Older Adults Undergoing Low- to Moderate-Risk Noncardiac Surgery—Secondary Analysis of a Prospective, Observer-Blinded, Randomized Clinical Trial

**DOI:** 10.3390/jcm13195946

**Published:** 2024-10-06

**Authors:** Alexander Taschner, Christian Reiterer, Edith Fleischmann, Barbara Kabon, Katharina Horvath, Nikolas Adamowitsch, David Emler, Thomas Christian, Nicole Hantakova, Beatrix Hochreiter, Laura Höfer, Magdalena List, Barbara Rossi, Florian W. Zenz, Giulia Zanvettor, Oliver Zotti, Melanie Fraunschiel, Alexandra Graf

**Affiliations:** 1 Department of Anesthesia, Intensive Care Medicine and Pain Medicine, Medical University of Vienna, Spitalgasse 23, 1090 Vienna, Austria; 2 IT Services and Strategic Information Management, Medical University of Vienna, Spitalgasse 23, 1090 Vienna, Austria; 3 Institute of Medical Statistics, Center for Medical Data Science, Medical University of Vienna, Spitalgasse 23, 1090 Vienna, Austria

**Keywords:** desflurane, sevoflurane, cardiac biomarkers, NT-proBNP, troponin T, copeptin, low- to moderate-risk noncardiac surgery, older adults

## Abstract

**Background/Objectives**: Previous preclinical studies have shown that desflurane might have the most significant cardioprotective effect of all volatile anesthetics. However, data regarding the cardioprotective effects of desflurane versus sevoflurane are lacking. Therefore, we evaluated the effect of the maintenance of anesthesia using desflurane versus sevoflurane on the postoperative maximum concentrations of cardiac biomarkers in older adults undergoing low- to moderate-risk noncardiac surgery. **Methods**: In this secondary analysis of a prospective randomized trial, we included all 190 older adults undergoing low- to moderate-risk noncardiac surgery. Patients were randomized to receive desflurane or sevoflurane for the maintenance of anesthesia. We administered desflurane or sevoflurane, aiming at a BIS value of 50 ± 5. The cardiac-specific biomarkers included troponin T, NT-proBNP, and copeptin, which were measured preoperatively, within one hour after surgery, and on the second postoperative day. **Results**: There were no significant differences between the desflurane and sevoflurane groups in the postoperative maximum concentrations of troponin T (11 ng.L^−1^ [8; 16] versus 13 ng.L^−1^ [9; 18]; *p* = 0.595), NT-proBNP (196 pg.mL^−1^ [90; 686] versus 253 pg.mL^−1^ [134; 499]; *p* = 0.288), or copeptin (19 pmol.L^−1^ [7; 58] versus 12 pmol.L^−1^ [6; 41]; *p* = 0.096). We also observed no significant differences in the troponin T, NT-proBNP, or copeptin concentrations between the desflurane and sevoflurane groups at any measured timepoint (all *p* > 0.05). **Conclusions**: In contrast to preclinical studies, we did not observe a significant difference in the postoperative maximum concentrations of cardiac biomarkers. It seems likely that desflurane does not exert significant clinical meaningful cardioprotective effects in older adults. Thus, our results do not support the use of desflurane in patients undergoing low- to moderate-risk noncardiac surgery.

## 1. Introduction

Cardiac complications after noncardiac surgery are some of the main reasons for postoperative morbidity and mortality. Specifically, older adults are at increased risk for developing postoperative myocardial injury after noncardiac surgery (MINS) [1], with an incidence of approximately 20% in patients over 65 years [1,2,3]. Age-related physiologic changes are associated with increased myocardial apoptosis or necrosis, leading to a reduction of vital cardiomyocytes, which might be the reason for the higher vulnerability to cardiac complications in older adults [4]. These effects could further explain why older adults are at increased risk for postoperative increases in the concentrations of cardiac biomarkers [1,5,6].

Several preclinical studies have indicated that preconditioning with volatile anesthetics exerts myocardial protection against ischemia [7,8]. Specifically, volatile anesthetics induce the opening of mitochondrial K_ATP_ channels, reducing mitochondrial Ca^2+^ overload, and thus, reducing myocardial cell death [7,9,10]. In contrast to sevoflurane, desflurane additionally inhibits the excessive opening of mitochondrial permeability transition pores, which potentially reduces mitochondria-driven cell death after ischemic reperfusion injury [8].

A study in a rabbit myocardium model showed that preconditioning with desflurane significantly attenuated infarct size after ischemia was induced by coronary artery occlusion as compared to sevoflurane [11]. However, in a clinical trial on patients undergoing on-pump cardiac surgery, there was no significant difference in the postoperative troponin T concentrations between the sevoflurane and desflurane groups [12]. Although the incidence of MINS, which is defined by increased troponin T concentrations, lies at approximately 20% [1,2,3], data regarding the effect of different volatile anesthetics on cardiac biomarkers after noncardiac surgery are still lacking.

Therefore, we performed a secondary analysis of our prospective, randomized clinical trial, in which we compared postoperative neurocognitive recovery in older adults between anesthesia with desflurane and that with sevoflurane [13]. In this secondary analysis, we evaluated the effects of desflurane versus sevoflurane on the maximum postoperative concentrations of troponin T. Furthermore, we compared the postoperative maximum NT-proBNP and copeptin concentrations between the groups.

## 2. Materials and Methods

### 2.1. Study Design and Participants

This pre-planned secondary analysis of a prospective randomized, observer-blinded, single-center clinical trial was conducted at the Medical University of Vienna after approval by the local Institutional Review Board (Ethics Committee of the Medical University of Vienna, Registration number: 1111/2022) and registration at ClinicalTrials.gov (NCT05331027) and at the European Clinical Trial Database (EudraCT 2022-000556-11). We evaluated patients scheduled for low- to moderate-risk surgery that was expected to last less than 2 h. Eligible patients were aged over 65 years for planned general anesthesia. We excluded patients who met one of the following criteria: (1) emergency surgery; (2) bariatric surgery; (3) dementia or neurologic disorder; (4) language, vision, or hearing impairments; (5) malignant hyperthermia; (6) structured muscle disease. We obtained written informed consent from all patients on the day before surgery.

### 2.2. Randomization and Masking

Shortly before the induction of anesthesia, we randomized patients at a 1:1 ratio using a web-based randomization program (Randomizer, Medical University of Graz, Graz, Austria; https://www.meduniwien.ac.at/randomizer/login, accessed on 28 September 2024) using permutated blocks. The patients were randomized to a desflurane group or a sevoflurane group respectively. The patients randomly assigned to the desflurane group received goal-directed administration of desflurane with the intraoperative goal of a bispectral index (BIS) of 50 ± 5. The patients randomly assigned to the sevoflurane group received goal-directed administration of sevoflurane with the intraoperative goal of a bispectral index (BIS) of 50 ± 5.

The attending anesthesiologists were not blinded toward the allocated group. The study personnel responsible for randomization were not involved in the outcome assessment. Both the patients and the blinded study personnel responsible for the postoperative outcome assessments were not informed about the randomized allocation. Postoperative visits of the study participants were only performed by the blinded study personnel. Furthermore, they had no access to the electronic anesthesia documentation.

### 2.3. Anesthesia Protocol

The induction and maintenance of anesthesia were standardized according to our study protocol [13]. After arriving to the operating room, all patients were monitored with an ECG, non-invasive blood pressure, SpO_2_, and BIS. For the induction of anesthesia, 1 µg.kg^−1^.body weight^−1^ (BW) remifentanil, 1 mg.kg^−1^.BW^−1^ propofol, and 0.6 mg.kg^−1^.BW^−1^ rocuronium were administered in all patients. Thereafter, intubation was performed if the BIS values were lower than 60. If the BIS values were higher than 60, a second bolus of 0.5 mg.kg^−1^.BW^−1^ propofol was administered. All patients received 4 mg of dexamethasone for the prophylaxis of postoperative nausea and vomiting.

We performed goal-directed administration of desflurane or sevoflurane in a mixed oxygen carrier gas using a fresh gas flow rate of 0.5 L.min^−1^ with an intraoperative goal of BIS 50 ± 5. The remifentanil infusion rate was started at 0.1 µg.kg^−1^.min^−1^. If the heart rate increased by 20% within 5 min and no changes in blood pressure or BIS values were observed, we increased the rate of remifentanil infusion by 0.05 µg.kg^−1^.min^−1^. Intraoperative vasopressor and fluid management was performed at the discretion of the attending anesthesiologist to maintain a minimum mean arterial pressure of 65 mmHg according to the clinical standard of care. We avoided the administration of atropine, scopolamine, and clonidine during surgery.

We stopped the administration of volatile agents and discontinued the infusion of remifentanil following skin closure. Thereafter, the fresh gas flow rate with 100% oxygen was set to the maximum. All patients received 200 mg sugammadex for the complete reversal of muscle relaxation. The patients having general or gynecologic surgery received 0.05 mg.kg^−1^.BW^−1^ piritramide after extubation. The patients having endoscopic urologic surgery received 0.025 mg.kg^−1^.BW^−1^ piritramide after extubation.

### 2.4. Measurements

We recorded the baseline demographic data of each patient, including their age, sex, weight, American Society of Anesthesiologists (ASA) physical status, comorbidities, long-term medication, and type of surgery. We further recorded the routine intraoperative data, including the duration of anesthesia and surgery, the amounts of fluid administration, medication, and vasopressors, the blood pressure at least every three minutes, the heart rate, and the continuous BIS values. We continuously recorded volatile anesthetic concentrations, converted these into minimum alveolar concentration (MAC) equivalents and expressed them as an age-adjusted fraction. Blinded research personnel drew all the study-specific blood samples. The troponin T, NT-proBNP, and copeptin levels were measured preoperatively shortly before the induction of anesthesia, within one hour after surgery, and on the second postoperative day in all patients if they were still in hospital. All laboratory measurements were performed by the Department of Laboratory Medicine at the Medical University of Vienna, Austria.

### 2.5. Data Management

Blinded research staff obtained all data. The electronic data were recorded in the data management software “Clincase”, version 2.7.0.12 (Quadratek Data Solutions Limited, Münzstraße 15, Berlin, Germany), hosted by the IT Services & Strategic Information Management, Medical University of Vienna, 1090 Vienna, Austria. The electronic case report form (eCRF) was designed by the science support work group “IT4Science”. Clincase provides advanced data management and monitoring, maintaining the GCP criteria. Accessible from multiple devices and locations, the web-based eCRF enables efficient user handling and, moreover, error avoidance and data preparation for statistical evaluation during or after the trial.

### 2.6. Statistical Analysis

The patients were analyzed on an intention-to-treat basis according to their randomized group. The continuous variables were summarized using the median and interquartile range separately for both groups. The categorical variables were summarized using absolute numbers and percentages for both groups. The maximum concentrations and concentrations for each timepoint separately of troponin T, NT-proBNP, and copeptin were first compared between the groups using Wilcoxon tests. We further performed univariable median regression models for the randomized group variable as well as for the confounding factors: age, body mass index (BMI), American Society of Anesthesiologists (ASA) physical status, duration of anesthesia, type of surgery, mean intraoperative blood pressure, long-term cardiovascular medication, cardiovascular comorbidities, and the respective baseline value of the cardiac biomarker. All significant variables in the univariable models were analyzed in a multivariable median regression model.

All *p*-values < 0.05 were considered statistically significant. Due to the exploratory character of this secondary analysis, no correction for multiplicity was performed. All analyses were performed using R, release 4.2.2.

### 2.7. Sample Size Estimation

The given sample size was based on the sample size estimation for the primary study outcome of postoperative neurocognitive recovery [13]. Therefore, no sample size calculation for the maximum concentrations of cardiac biomarkers was performed. However, we previously observed that troponin T concentrations after noncardiac surgery increased up to 18 ng.L^−1^ (standard deviation: 7 ng.L^−1^) [2]. We assumed a similar postoperative increase in the sevoflurane group. Since this was a pragmatic clinical trial evaluating two different routinely used anesthetics, a relatively lower increase of 20% (to 14.4 ng.L^−1^) in the desflurane group was defined as clinically meaningful. A total of 65 patients per group was needed to achieve at least 80% power to detect the assumed difference between the groups using a Wilcoxon rank-sum test with a two-sided significance level of 0.05. We, therefore, included all 190 patients originally enrolled in the trial to ensure sufficient power for the secondary analysis [13].

## 3. Results

### 3.1. Patient Characteristics

**Figure 1:**
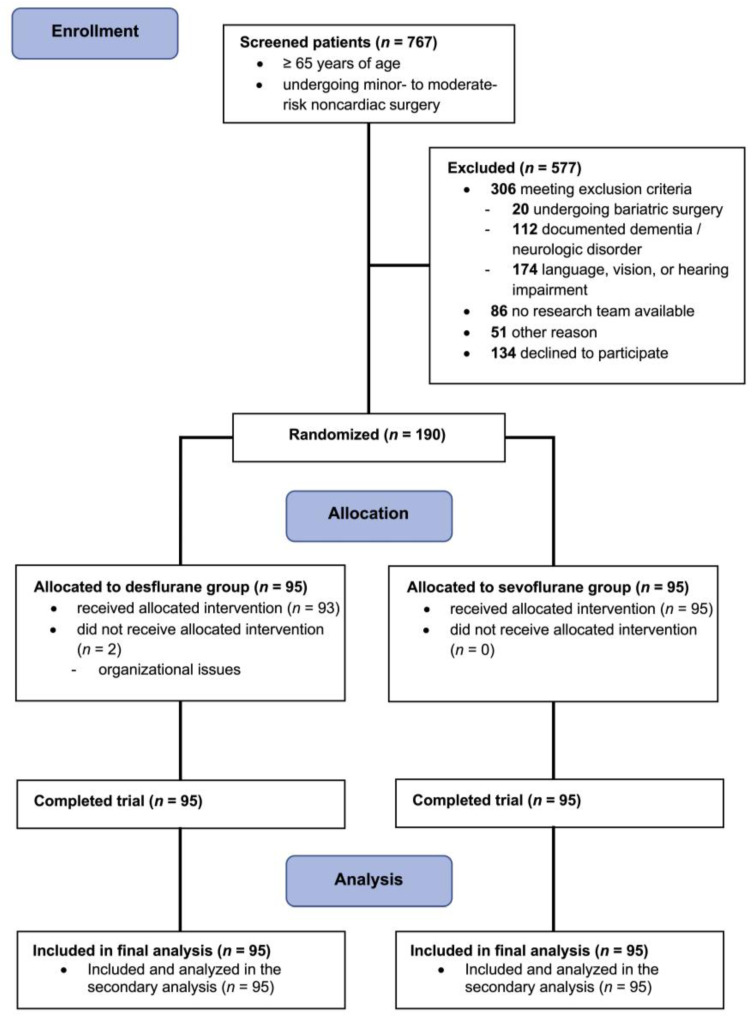
Patient flow diagram; design and form in concordance with the 2010 CONSORT guidelines [14].

We included 190 older adults undergoing low- to moderate-risk noncardiac surgery between May 2022 and April 2023 at the Medical University of Vienna in the main trial [13]. Enrolment was ceased after reaching our target sample size of 190 patients. Two patients in the desflurane group received sevoflurane due to organizational issues (Figure 1). We included all patients who were included in the main trial in this secondary analysis. The baseline characteristics were similar between the groups (Table 1).

Intraoperative minimum alveolar concentration (MAC) fractions were significantly lower in the desflurane group (0.48) as compared to the sevoflurane group (0.58; *p* < 0.001). The patients in the sevoflurane group had significantly lower intraoperative heart rates (58 beats.m^−1^ [54; 64] versus 61 beats.min^−1^ [56; 67]; *p* = 0.026) and mean arterial pressure levels (77 mmHg [72; 82] versus 80 mmHg [75; 87]; *p* = 0.010) as compared to the desflurane group. The inspiratory and expiratory concentrations of desflurane were significantly higher than those of sevoflurane (Table 2). Otherwise, there were no significant differences in the intraoperative parameters, including the type of surgery, duration of surgery and anesthesia, and the fluid and vasopressor management between the groups (Table 2).

### 3.2. Troponin T

**Figure 2:**
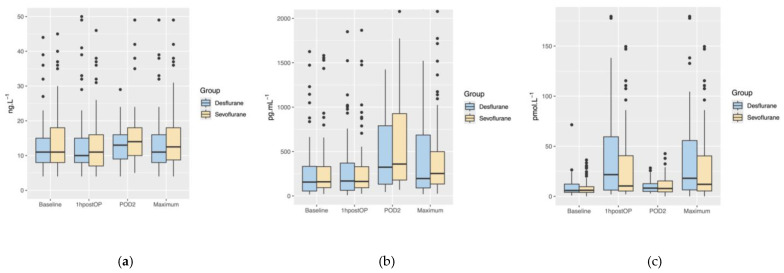
Boxplots showing the perioperative time course and postoperative maximum concentrations of troponin T (**a**), NT-proBNP (**b**), and copeptin (**c**) separately for the desflurane group (blue) and the sevoflurane group (yellow). Boxplots demonstrate medians and interquartile ranges, dots represent outliers. 1hpostOP, within one hour after surgery; POD2, postoperative day 2.

The postoperative maximum troponin T concentrations did not differ significantly between the desflurane group (11 ng.L^−1^ [IQR of 8; 16]) and the sevoflurane group (13 ng.L^−1^ [IQR of 9; 18]) (*p* = 0.595) (Figure 2a). We observed no significant difference in the troponin T concentrations between the desflurane and sevoflurane groups at any measured timepoint (all *p* > 0.05) (Table 3).

In the univariable median regression model, the randomized group did not show a significant effect on the postoperative maximum troponin T concentrations (effect estimate: 1.746; 95%CI: −1.331–4.823; *p* = 0.267). Of all the covariables included in the median regression models, only the baseline troponin T concentrations remained significant in the multivariable median regression model (*p* < 0.001). The detailed results of the univariable and multivariable median regression models are presented in Appendix A, Table A1.

### 3.3. NT-proBNP

The postoperative maximum NT-proBNP concentrations did not differ significantly between the desflurane group (196 pg.mL^−1^ [IQR of 90; 686]) and the sevoflurane group (253 pg.mL^−1^ [IQR of 134; 499]) (*p* = 0.288) (Figure 2b). We observed no significant difference in the NT-proBNP concentrations between the desflurane and sevoflurane groups at any measured timepoint (all *p* > 0.05) (Table 3).

In the univariable median regression model, the randomized group did not show a significant effect on the postoperative maximum NT-proBNP concentrations (effect estimate: 56.292; 95%CI: −43.716–156.300; *p* = 0.271). Of all pre-defined covariables included in the median regression models, only the baseline NT-proBNP concentrations remained significant in the multivariable median regression model (*p* = 0.036). The detailed results of the univariable and multivariable median regression models are presented in Appendix A, Table A2.

### 3.4. Copeptin

The postoperative maximum copeptin concentrations did not differ significantly between the desflurane group (18.6 pmol.L^−1^ [IQR of 6.8; 57.6]) and the sevoflurane group (12.2 pmol.L^−1^ [IQR of 6.0; 40.6) (*p* = 0.096) (Figure 2c). We observed no significant difference in the copeptin concentrations between the desflurane and sevoflurane groups at any measured timepoint (all *p* > 0.05) (Table 3).

In the univariable median regression model, the randomized group did not show a significant effect on the postoperative maximum copeptin concentrations (effect estimate: −6.325; 95% CI: −16.579–3.929; *p* = 0.228). Of all pre-defined covariables included in the median regression models, only the baseline copeptin concentrations (*p* < 0.001) and duration of anesthesia (*p* = 0.007) remained in the multivariable median regression model. The detailed results of the univariable and multivariable median regression models are presented in Appendix A, Table A3.

## 4. Discussion

General anesthesia with desflurane did not significantly reduce the postoperative troponin T, NT-proBNP, and copeptin concentrations in older adults undergoing low- to moderate-risk noncardiac surgery, as compared to sevoflurane.

Some relatively small preclinical studies have indicated distinctive cardioprotective effects of desflurane during ischemia/reperfusion [7,8,10,11]. Compared to all other volatile anesthetics, desflurane has also led to the most significant reduction of myocardial infarct size after coronary artery occlusion [11]. In contrast to these preclinical studies, cardioprotective effects could not be observed in clinical studies. In a randomized trial, De Hert et al. evaluated the effects of sevoflurane and desflurane versus propofol on the postoperative troponin T concentrations in patients undergoing on-pump coronary artery surgery [12]. They found no significant difference in the postoperative troponin T concentrations between the groups [12]. We also did not observe a significant difference in any of the measured cardiac biomarkers. One explanation for the negative results in clinical studies might be the differences in the study settings. In preclinical studies, volatile anesthetics were tested in healthy myocardium for myocardial preconditioning, which means that anesthetics were administered before the ischemic event was initiated [7,8,10,11]. In contrast, in clinical studies, volatile anesthetics were administered in patients with and without signs of cardiac ischemia. Specifically, in cardiac surgery, ischemic events often happened before surgery and are, thus, the indication for the surgical procedure per se [15,16]. Therefore, the effect of myocardial preconditioning was negligible, and desflurane anesthetics might not exert the same cardioprotective effects as observed in preclinical studies.

A significant difference between our study and previous clinical trials in patients undergoing on-pump coronary artery surgery is the perioperative ischemic times. While on-pump coronary artery bypass graft surgery is associated with induced myocardial ischemia due to myocardial damage by surgery per se [17] perioperative myocardial injury during noncardiac surgery is largely attributed to perioperative hemodynamic perturbations, leading to oxygen supply–demand mismatches [18,19]. In this context, it is important to note that on-pump cardiac surgery per se is associated with an increase in postoperative troponin concentrations due to surgical injury to myocardial tissue. In contrast, postoperative elevations of troponin T concentrations are specific for ischemic myocardial damage. In patients undergoing noncardiac surgery, peak postoperative high-sensitivity troponin T concentrations ≥ 14 ng.L^−1^ are independently associated with higher 30-day mortality [3]. In contrast, in patients undergoing on-pump coronary artery bypass graft surgery, only peak postoperative high-sensitivity troponin T concentrations ≥ 400 ng.L^−1^ were independently significantly associated with a higher 30-day mortality [17]. Based on this, comparisons of our results and the results of previous studies, which investigated the effects of desflurane versus sevoflurane in patients undergoing on-pump cardiac surgery need to be viewed with this limitation. Further studies in patients undergoing noncardiac surgery are, therefore, needed.

Another explanation for the inconsistent results between preclinical and clinical studies are the administered concentrations of volatile anesthetics. Several preclinical studies have indicated a dose-dependent cardioprotective effect of volatile anesthetics [7,10]. Specifically, the most effective cardioprotection was observed at expiratory concentrations of desflurane of 6% [10]. In contrast, De Hert et al. administered volatile anesthetics to aim at 0.5 MAC [12]. Similarly, patients in our study also had intraoperative mean MAC fractions of 0.48 MAC in the desflurane group and 0.58 in the sevoflurane group. Therefore, it is likely that in clinical studies, the intraoperatively used concentrations of desflurane were too low to exert the previously observed cardioprotective effects.

Cardioprotection of desflurane against ischemic injury in preclinical studies was evaluated by assessing in vitro specimens of myocardium before and after ischemia, which is not translatable to clinical practice. However, large observational studies have shown that the postoperative concentrations of troponin T, NT-proBNP, and copeptin are independent predictors of cardiovascular morbidity and mortality [1,3,20,21]. Therefore, these biomarkers are already established in clinical practice, and perioperative measurements are recommended in guidelines [22]. We, therefore, chose to measure these biomarkers to assess the cardioprotective effects of desflurane as compared to sevoflurane.

There are also several studies revealing harmful effects on the cardiovascular system when desflurane is administered [23,24,25,26,27]. A unique feature of desflurane, as compared to sevoflurane and isoflurane, is a significant increase in sympathetic nervous activity, which is mainly explained by direct stimulation of the medullary centers [23,24,25,26,27]. In detail, desflurane leads to a dose-dependent significant increase in the heart rate, mean arterial pressure, plasma catecholamine, and plasma arginine–vasopressin (AVP) concentrations as compared to isoflurane [26]. Copeptin is co-released with AVP and is a surrogate parameter of AVP [28,29]. Interestingly, in our study, the copeptin concentrations did not differ significantly between the groups. However, we did observe statistically significant higher intraoperative heart rate and mean arterial pressure levels in the desflurane group, but this was not clinically meaningful. An explanation for this might be that we used propofol for the induction of anesthesia, which is known to significantly blunt hemodynamic effects caused by desflurane [26,30]. Another explanation for our hemodynamic observations could be that we performed BIS-guided anesthesia, which resulted in relatively low MAC values in both groups. Ebert et al. reported that the highest heart rate and sympathetic nerve activity was observed at 1.5 MAC and was dose-dependent [27], which, in fact, was nearly three times as high as in our study.

This study has some limitations. Firstly, this is a secondary analysis of a prospective trial, and the sample size was powered to detect a difference in the postoperative neurocognitive recovery between the desflurane and the sevoflurane groups [13]. Associations may, therefore, be interpreted only in an exploratory way.

Secondly, we only included patients undergoing low- to moderate-risk noncardiac surgery lasting a maximum of two hours. Thus, our results cannot be extrapolated to patients undergoing major noncardiac surgery. Since it is known that major noncardiac surgery is associated with a higher myocardial strain, specifically in patients with cardiovascular risk [31], significant effects in these patients cannot be ruled out. Therefore, further studies must be performed to evaluate a possible cardioprotective effect of desflurane. Third, we only measured the cardiac biomarkers immediately after the end of surgery and on the second postoperative day. Previous studies have shown that troponin T and NT-proBNP concentrations peaked on the second postoperative day. Furthermore, we previously showed that copeptin concentrations peaked immediately after the end of surgery [2,3,32]. Nevertheless, it is still possible that the patients had higher troponin T, NT-proBNP, and copeptin concentrations on the first postoperative day, which would have been missed in our results. Furthermore, a major limitation is that we did not perform the measurements of the cardiac biomarkers on the second postoperative day in the patients who were discharged. Detailed descriptions of the timepoints of hospital discharge have been reported within the main publication [13]. A total of 49 patients in the desflurane group and 51 patients in the sevoflurane group were still in the hospital on the second postoperative day, and the cardiac biomarker concentrations were only measured in these patients, which showed no significant differences between the groups. Nevertheless, it cannot be ruled out that the postoperative maximum concentrations could have differed significantly if the biomarkers were measured in all patients on the second postoperative day.

In summary, we did not observe a significant effect of desflurane versus sevoflurane on the postoperative maximum concentrations of troponin T, NT-proBNP, and copeptin in older adults undergoing low- to moderate-risk noncardiac surgery. Based on our findings, it seems likely that the clinical effects of desflurane versus sevoflurane on perioperative cardiac-specific biomarkers in this patient population are negligible.

## Figures and Tables

**Table 1:** Patient baseline characteristics. Summary characteristics are presented as counts (percentages) of patients or medians [25th percentile; 75th percentile].

	Desflurane (*n* = 95)		Sevoflurane (*n* = 95)	
Age, yrs	73	[70; 80]	73	[70; 78]
Height, cm	170	[164; 176]	170	[161; 178]
Weight, kg	75	[66; 86]	77	[66; 87]
BMI, kg.m^−2^	26.0	[23.5; 30.2]	26.3	[23.8; 28.7]
Sex, *n* (%)				
Female	44	(46.3)	46	(48.4)
Male	51	(53.7)	49	(51.6)
ASA, *n* (%)				
I–II	52	(54.7)	49	(51.6)
III	43	(45.3)	46	(48.4)
Comorbidities, *n* (%)				
Hypertension	55	(57.9)	57	(60.0)
Coronary artery disease	7	(7.3)	9	(9.5)
Peripheral artery disease	2	(2.1)	4	(4.2)
Carotid artery stenosis	6	(6.3)	10	(10.5)
TIA/Stroke	6	(6.3)	3	(3.2)
Atrial fibrillation	21	(22.1)	12	(12.6)
COPD	16	(16.8)	11	(11.6)
Diabetes	18	(18.9)	20	(21.1)
Hyperlipidemia	33	(34.7)	31	(32.6)
History of smoking	17	(17.9)	6	(6.3)
Long-term medication, *n* (%)				
Beta blockers	30	(31.6)	30	(31.6)
ACE-I/ARB	45	(47.4)	47	(49.5)
Ca^2+^ channel blockers	21	(22.1)	22	(23.2)
Diuretics	16	(16.8)	24	(25.3)
Statins	37	(38.9)	31	(32.6)
Thienopyridines/ASA	20	(21.1)	22	(23.2)
Oral anticoagulant	19	(20.0)	15	(15.8)
Metformin	9	(9.5)	15	(15.8)
Type of Surgery, (%)				
Minor to moderate general	12	(12.6)	7	(7.4)
Thyroidectomy	3	(25.0)	2	(28.6)
Parathyroidectomy	2	(16.7)	1	(14.3)
Lap. cholecystectomy	2	(16.7)	1	(14.3)
Inguinal hernia surgery	2	(16.7)	0	(0)
Lap. fundoplication surgery	0	(0)	1	(14.3)
Ileostomy surgery	2	(16.7)	0	(0)
Other	1	(8.3)	2	(28.6)
Minor to moderate urologic	53	(55.8)	49	(51.6)
TUR-B	35	(66.0)	37	(75.5)
TUR-P	11	(20.8)	7	(14.3)
Other	7	(13.2)	5	(10.2)
Minor to moderate gynecologic	30	(31.6)	39	(41.1)
Lap. ovariectomy	1	(3.3)	1	(2.6)
Lap. salpingectomy	4	(13.3)	5	(12.8)
Lap. hysterectomy	6	(20.0)	4	(10.3)
Mamma resection	19	(63.3)	29	(74.4)

BMI, body mass index; ASA, American Society of Anesthesiologists physical status; TIA, transient ischemic attack; COPD, chronic obstructive pulmonary disease; ACE-I, angiotensin-converting enzyme inhibitor; ARB, angiotensin receptor blocker.

**Table 2:** Perioperative characteristics. Summary characteristics of perioperative variables are presented as the medians [25th percentile; 75th percentile] or counts (percentage) of patients. All *p*-values are for Wilcoxon tests.

	Desflurane (*n* = 95)		Sevoflurane (*n* = 95)		*p*-Value
Duration of anesthesia, min	83	[54; 109]	80	[58; 106]	0.868
Duration of surgery, min	55	[28; 82]	53	[33; 75]	0.746
Duration transfer to PACU, min	8	[7; 9]	8	[6; 9]	0.888
Intraoperative management					
Crystalloids, ml	500	[500; 1000]	500	[500; 1000]	0.215
Propofol, mg	90	[70; 112]	80	[70; 105]	0.505
Remifentanil, mg	0.67	[0.41; 1.02]	0.61	[0.43; 0.86]	0.498
Rocuronium, mg	50	[40; 60]	40	[40;50]	0.127
Phenylephrine, mg	0.14	[0.08; 0.24]	0.22	[0.1; 0.4]	0.188
Etilefrine, mg	4.0	[2; 5.5]	2.0	[2.0; 3.5]	0.247
Piritramide, mg	3.0	[2.0; 3.8]	3.0	[2.2; 3.5]	0.436
HR, beats.min^−1^	61	[56; 67]	58	[54; 64]	0.026
MAP, mmHg	80	[75; 87]	77	[72; 82]	0.010
etCO_2_, mmHg	35	[33; 37]	35	[33; 37]	0.62
FiO_2_, %	50	[43; 57]	50	[45; 57]	0.332
Insp. agent, %	3.4	[2.9; 3.8]	1.3	[1.1; 1.4]	<0.001
Expir. agent, %	2.9	[2.4; 3.3]	1.0	[0.9; 1.1]	<0.001
Ta, °C	36.4	[36.1; 36.7]	36.3	[36.0; 36.5]	0.304
BIS	51	[45; 55]	52	[48; 56]	0.071
At PACU					
Piritramide, mg	3.7	[0.0; 7.5]	3.0	[0.0; 6.0]	0.395
Diclofenac, no. (%)	18	(19.0)	11	(11.6)	0.158
Metamizole, no. (%)	41	(43.2)	33	(34.7)	0.234
Droperidol, no. (%)	14	(14.7)	9	(9.5)	0.266
Ondansetron, no. (%)	9	(9.5)	10	(10.5)	0.809
HR, beats.min^−1^	72	[64; 82]	70	[63; 80]	0.363
MAP, mmHg	111	[101; 120]	108	[99; 116]	0.098

PACU, post-anesthesia care unit; HR, heart rate; MAP, mean arterial pressure; etCO_2_, expiratory carbon dioxide; FiO_2_, fraction of inspired oxygen; Ta, temperature; BIS, bispectral index; Insp. agent, inspiratory agent concentration; Expir. agent, expiratory agent concentration.

**Table 3:** Perioperative concentrations of cardiac biomarkers. Summary characteristics are presented as medians [25th quartile; 75th quartile]. All *p*-values are for Wilcoxon tests.

	Desflurane (*n* = 95)		Sevoflurane (*n* = 95)		*p*-Value
Troponin T, ng.L^−1^ (n desflurane/n sevoflurane)					
Baseline, (89/90)	11	[8; 15]	11	[8; 18]	0.529
Postoperative, (87/89)	10	[8; 15]	11	[7; 16]	0.539
Postoperative Day 2, (49/51)	13	[9; 16]	14	[10; 18]	0.445
Maximum, (89/92)	11	[8; 16]	13	[9; 18]	0.595
NT-proBNP, pg.mL^−1^ (n desflurane/n sevoflurane)					
Baseline, (89/90)	156	[55; 333]	150	[93; 321]	0.314
Postoperative, (87/89)	160	[64; 370]	165	[93; 334]	0.465
Postoperative Day 2, (49/51)	328	[135; 784]	362	[182; 927]	0.393
Maximum, (89/92)	196	[90; 686]	253	[134; 499]	0.288
Copeptin, pmol.L^−1^ (n desflurane/n sevoflurane)					
Baseline, (86/87)	5.9	[4.0; 12.7]	5.9	[3.8; 9.5]	0.882
Postoperative, (85/86)	21.7	[6.3; 59.5]	10.4	[5.4; 40.6]	0.112
Postoperative Day 2, (45/48)	8.2	[5.0; 12.4]	7.9	[4.5; 15.4]	0.738
Maximum, (87/90)	18.6	[6.8; 57.6]	12.2	[6.0; 40.6]	0.096

## Data Availability

The data presented in this secondary analysis are available upon request from the corresponding author.

## Appendix A

**Table A1:** Univariable and multivariable median regression models for postoperative maximum troponin T concentrations.

Variable	Comparison	Estimated Effect	Lower 95% CL	Upper 95% CL	*p*-Value
Univariable median regression analysis					
Randomization group	Sevoflurane vs. Desflurane	1.746	−1.331	4.823	0.267
Age		0.615	0.459	0.772	<0.001
BMI		0.027	−0.236	0.290	0.840
Sex	Male vs. Female	6.000	3.865	8.135	<0.001
ASA	III vs. I–II	5.000	2.502	7.498	<0.001
Baseline troponin T		0.941	0.858	1.025	<0.001
Duration of anesthesia		0.012	−0.012	0.036	0.329
Type of surgery					
	Urologic vs. General	6.094	1.447	10.742	0.011
	Gynecologic vs. General	0.764	−3.729	5.257	0.739
	Gynecologic vs. Urologic	−5.330	−7.534	−3.127	<0.001
Intraoperative MAP		0.167	0.008	0.327	0.041
Beta-blocker	Yes vs. No	4.000	0.892	7.108	0.013
Ca^2+^ channel blocker	Yes vs. No	2.556	−0.516	5.628	0.105
Aspirin	Yes vs. No	4.000	0.094	7.906	0.046
Statin	Yes vs. No	4.000	0.557	7.443	0.024
Xa inhibitor	Yes vs. No	4.000	−1.045	9.045	0.122
ACE inhibitor	Yes vs. No	5.000	2.564	7.436	<0.001
Hypertension	Yes vs. No	5.000	2.702	7.298	<0.001
Myocardial infarction	Yes vs. No	12.000	0.304	23.696	0.046
Diabetes II	Yes vs. No	3.502	0.042	6.962	0.049
Multivariable median regression analysis					
Age		0.017	−0.112	0.147	0.795
Sex	Male vs. Female	0.309	−1.598	2.216	0.751
ASA	III vs. I–II	1.323	−0.107	2.752	0.072
Baseline troponin T		0.895	0.762	1.028	<0.001
Type of surgery					
	Urologic vs. General	0.130	−1.845	2.104	0.898
	Gynecologic vs. General	−0.586	−2.260	1.087	0.493
	Gynecologic vs. Urologic	−0.716	−2.587	1.155	0.454
Intraoperative MAP		0.024	−0.052	0.099	0.536
Beta-blocker	Yes vs. No	1.101	−0.361	2.563	0.142
Aspirin	Yes vs. No	0.073	−1.785	1.931	0.938
Statin	Yes vs. No	−0.322	−1.855	1.210	0.681
ACE inhibitor	Yes vs. No	−0.657	−2.458	1.144	0.476
Hypertension	Yes vs. No	0.273	−1.567	2.113	0.772
Myocardial infarction	Yes vs. No	0.629	−2.743	4.001	0.715
Diabetes II	Yes vs. No	−0.308	−2.000	1.384	0.722

CL, confidence limit; BMI, body mass index; ASA, American Society of Anesthesiologists physical status; MAP, mean arterial pressure; ACE, angiotensin converting enzyme.

**Table A2:** Univariable and multivariable median regression models for postoperative maximum NT-proBNP concentrations.

Variable	Comparison	Estimated Effect	Lower 95% CL	Upper 95% CL	*p*-Value
Univariable median regression analysis					
Randomization group	Sevoflurane vs. Desflurane	56.292	−43.716	156.300	0.271
Age		27.600	13.646	41.554	<0.001
BMI		−0.073	−7.862	6.316	0.831
Sex	Male vs. Female	−35.961	−125.105	53.184	0.43
ASA	III vs. I–II	143.000	−57.748	343.748	0.164
Baseline NT-proBNP		1.101	0.444	1.759	<0.001
Duration of anesthesia		1.222	−0.131	2.575	0.078
Type of surgery					
	Urologic vs. General	68.026	−88.270	224.322	0.395
	Gynecologic vs. General	131.938	−28.675	292.551	0.109
	Gynecologic vs. Urologic	63.912	−43.352	171.175	0.244
Intraoperative MAP		6.237	−0.516	12.991	0.072
Beta-blocker	Yes vs. No	282.844	77.135	488.554	0.008
Ca^2+^ channel blocker	Yes vs. No	23.319	−98.258	144.896	0.707
Aspirin	Yes vs. No	−8.257	−194.771	178.257	0.931
Statin	Yes vs. No	20.000	−100.271	140.271	0.745
Xa inhibitor	Yes vs. No	549.024	193.549	904.499	0.003
ACE inhibitor	Yes vs. No	77.854	−61.885	217.593	0.276
Hypertension	Yes vs. No	70.461	−62.878	203.800	0.302
Myocardial infarction	Yes vs. No	213.847	−267.607	695.302	0.385
Diabetes II	Yes vs. No	72.683	−156.233	301.600	0.535
Multivariable median regression analysis					
Age		2.326	−7.423	12.075	0.641
Baseline NT-proBNP		1.047	0.076	2.017	0.036
Beta-blocker	Yes vs. No	29.127	−93.321	151.576	0.642
Xa inhibitor	Yes vs. No	−18.641	−185.280	148.358	0.829

CL, confidence limit; BMI, body mass index; ASA, American Society of Anesthesiologists physical status; MAP, mean arterial pressure; ACE, angiotensin converting enzyme.

**Table A3:** Univariable and multivariable median regression models for postoperative maximum copeptin concentrations.

Variable	Comparison	Estimated Effect	Lower 95% CL	Upper 95% CL	*p*-Value
Univariable median regression analysis					
Randomization group	Sevoflurane vs. Desflurane	−6.325	−16.579	3.929	0.228
Age		0.697	−0.137	1.531	0.103
BMI		−0.012	−0.771	0.747	0.976
Sex	Male vs. Female	3.052	−6.471	12.574	0.531
ASA	III vs. I–II	6.710	−3.293	16.713	0.190
Baseline copeptin		1.336	0.958	1.714	<0.001
Duration of anesthesia		0.148	0.046	0.250	0.005
Type of surgery					
	Urologic vs. General	−10.598	−30.838	9.642	0.306
	Gynecologic vs. General	−12.455	−34.511	9.601	0.270
	Gynecologic vs. Urologic	−1.857	−10.479	6.766	0.673
Intraoperative MAP		0.131	−0.268	0.529	0.521
Beta-blocker	Yes vs. No	6.174	−5.330	17.678	0.294
Ca^2+^ channel blocker	Yes vs. No	−0.120	−12.316	12.077	0.985
Aspirin	Yes vs. No	3.700	−14.218	21.619	0.686
Statin	Yes vs. No	−2.353	−9.962	5.255	0.545
Xa inhibitor	Yes vs. No	2.891	−5.488	11.270	0.500
ACE inhibitor	Yes vs. No	−8.433	−19.299	2.432	0.130
Hypertension	Yes vs. No	−1.941	−12.881	8.999	0.728
Myocardial infarction	Yes vs. No	8.680	−4.898	22.258	0.212
Diabetes II	Yes vs. No	10.180	−1.263	21.623	0.083
Multivariable median regression analysis					
Baseline copeptin		1.217	0.985	1.450	<0.001
Duration of anesthesia		0.130	0.037	0.223	0.007

CL, confidence limit; BMI, body mass index; ASA, American Society of Anesthesiologists physical status; MAP, mean arterial pressure; ACE, angiotensin converting enzyme.

## References

[1] Botto F., Alonso-Coello P. (2014). Myocardial injury after noncardiac surgery: A large, international, prospective cohort study establishing diagnostic criteria, characteristics, predictors and 30-day outcomes. Anesthesiology.

[2] Reiterer C., Kabon B., Taschner A., von Sonnenburg M.F., Graf A., Adamowitsch N., Starlinger P., Goshin J., Fraunschiel M., Fleischmann E. (2021). Perioperative supplemental oxygen and NT-proBNP concentrations after major abdominal surgery—A prospective randomized clinical trial. J. Clin. Anesth..

[3] Devereaux P.J., Biccard B.M., Sigamani A., Xavier D., Chan M.T.V., Srinathan S.K., Walsh M., Abraham V., Pearse R., Writing Committee for the VISION Study Investigators (2017). Association of postoperative high-sensitivity troponin levels with myocardial injury and 30-day mortality among patients undergoing noncardiac surgery. JAMA-J. Am. Med. Assoc..

[4] Nakou E.S., Parthenakis F.I., Kallergis E.M., Marketou M.E., Nakos K.S., Vardas P.E. (2016). Healthy aging and myocardium: A complicated process with various effects in cardiac structure and physiology. Int. J. Cardiol..

[5] Mauermann E., Bolliger D., Seeberger E., Puelacher C., Corbiere S., Filipovic M., Seeberger M., Mueller C., Buse G.L. (2016). Incremental value of preoperative copeptin for predicting myocardial injury. Anesth. Analg..

[6] Rodseth R.N., Biccard B.M., Chu R., Buse G.A.L., Thabane L., Bakhai A., Bolliger D., Cagini L., Cahill T.J., Cardinale D. (2013). Postoperative B-type natriuretic peptide for prediction of major cardiac events in patients undergoing noncardiac surgery: Systematic review and individual patient meta-analysis. Anesthesiology.

[7] Hanouz J.-L., Yvon A., Massetti M., Lepage O., Babatasi G., Khayat A., Bricard H., Gérard J.-L. (2002). Mechanisms of Desflurane-induced Preconditioning in Isolated Human Right Atria In Vitro. Anesthesiology.

[8] Piriou V., Chiari P., Gateau-Roesch O., Argaud L., Muntean D., Salles D., Loufouat J., Gueugniaud P.-Y., Lehot J.-J., Ovize M. (2004). Desflurane-induced Preconditioning Alters Calcium-induced Mitochondrial Permeability Transition. Anesthesiology.

[9] Peyronnet R., Nerbonne J.M., Kohl P. (2016). Cardiac Mechano-Gated Ion Channels and Arrhythmias. Circ. Res..

[10] Lemoine S., Beauchef G., Zhu L., Renard E., Lepage O., Massetti M., Khayat A., Galera P., Gérard J.-L., Hanouz J.-L. (2008). Signaling Pathways Involved in Desflurane-induced Postconditioning in Human Atrial Myocardium In Vitro. Anesthesiology.

[11] Piriou V., Chiari P., Lhuillier F., Bastien O., Loufoua J., Raisky O., David J., Ovize M., Lehot J.J. (2002). Pharmacological preconditioning: Comparison of desflurane, sevoflurane, isoflurane and halothane in rabbit myocardium. Br. J. Anaesth..

[12] De Hert S., Vlasselaers D., Barbé R., Ory J., Dekegel D., Donnadonni R., Demeere J., Mulier J., Wouters P. (2009). A comparison of volatile and non volatile agents for cardioprotection during on-pump coronary surgery. Anaesthesia.

[13] Taschner A., Fleischmann E., Horvath K., Adamowitsch N., Emler D., Christian T., Hantakova N., Hochreiter B., Höfer L., List M. (2024). Desflurane versus sevoflurane anesthesia and postoperative recovery in older adults undergoing minor- to moderate-risk noncardiac surgery—A prospective, randomized, observer-blinded, clinical trial. J. Clin. Anesth..

[14] Schulz K.F., Altman D.G., Moher D. (2011). CONSORT 2010 statement: Updated guidelines for reporting parallel group randomised trials. Int. J. Surg..

[15] Guarracino F., Landoni G., Tritapepe L., Pompei F., Leoni A., Aletti G., Scandroglio A.M., Maselli D., De Luca M., Marchetti C. (2006). Myocardial Damage Prevented by Volatile Anesthetics: A Multicenter Randomized Controlled Study. J. Cardiothorac. Vasc. Anesth..

[16] Landoni G., Lomivorotov V.V., Nigro Neto C., Monaco F., Pasyuga V.V., Bradic N., Lembo R., Gazivoda G., Likhvantsev V.V., Lei C. (2019). Volatile Anesthetics versus Total Intravenous Anesthesia for Cardiac Surgery. N. Engl. J. Med..

[17] Nellipudi J.A., Baker R.A., Dykes L., Krieg B.M., Bennetts J.S. (2021). Prognostic value of high-sensitivity Troponin T after on-pump coronary artery bypass graft surgery. Heart Lung Circ..

[18] Priebe H.J. (2004). Triggers of perioperative myocardial ischaemia and infarction. Br. J. Anaesth..

[19] Devereaux P.J., Goldman L., Cook D.J., Gilbert K., Leslie K., Guyatt G.H. (2005). Perioperative cardiac events in patients undergoing noncardiac surgery: A review of the magnitude of the problem, the pathophysiology of the events and methods to estimate and communicate risk. Can. Med. Assoc. J..

[20] Duceppe E., Patel A., Chan M.T., Berwanger O., Ackland G., Kavsak P.A., Rodseth R., Biccard B., Chow C.K., Borges F.K. (2019). Preoperative N-Terminal Pro-B-Type Natriuretic Peptide and Cardiovascular Events After Noncardiac Surgery: A Cohort Study. Ann. Intern. Med..

[21] Jarai R., Mahla E., Perkmann T., Jarai R., Archan S., Tentzeris I., Huber K., Metzler H. (2011). Usefulness of pre-operative copeptin concentrations to predict post-operative outcome after major vascular surgery. Am. J. Cardiol..

[22] Halvorsen S., Mehilli J., Cassese S., Hall T.S., Abdelhamid M., Barbato E., De Hert S., de Laval I., Geisler T., Hinterbuchner L. (2022). 2022 ESC Guidelines on cardiovascular assessment and management of patients undergoing non-cardiac surgery. Eur. Heart J..

[23] Ebert T.J., Muzi M., Lopatka C.W. (1995). Neurocirculatory responses to sevoflurane in humans—A comparison to desflurane. Anesthesiology.

[24] Muzi M., Ebert T.J., Hope W.G., Robinson B.J., Bell L.B. (1996). Site(s) mediating sympathetic activation with desflurane. Anesthesiology.

[25] Ebert T.J., Perez F., Uhrich T.D., Deshur M.A. (1998). Desflurane-mediated sympathetic activation occurs in humans despite preventing hypotension and baroreceptor unloading. Anesthesiology.

[26] Welskopf R.B., Moore M.A., Eger E.I., Noorani M., McKay L., Chortkoff B., Hart P.S., Damask M. (1994). Rapid increase in desflurane concentration is associated with greater transient cardiovascular stimulation than with rapid increase in isoflurane concentration in humans. Anesthesiology.

[27] Ebert T.J., Muzi M. (1993). Sympathetic hyperactivity during desflurane anesthesia in healthy volunteers—A comparison with isoflurane. Anesthesiology.

[28] Lipinski M.J., Escárcega R.O., D’Ascenzo F., Magalhães M.A., Baker N.C., Torguson R., Chen F., Epstein S.E., Miró O., Llorens P. (2014). A systematic review and collaborative meta-analysis to determine the incremental value of copeptin for rapid rule-out of acute myocardial infarction. Am. J. Cardiol..

[29] Bolignano D., Cabassi A., Fiaccadori E., Ghigo E., Pasquali R., Peracino A., Peri A., Plebani M., Santoro A., Settanni F. (2014). Copeptin (CTproAVP), a new tool for understanding the role of vasopressin in pathophysiology. Clin. Chem. Lab. Med..

[30] Lopatka C.W., Muzi M., Ebert T.J. (1999). Propofol, but not etomidate, reduces desflurane-mediated sympathetic activation in humans. Can. J. Anaesth..

[31] van Waes J.A., Nathoe H.M., de Graaff J.C., Kemperman H., de Borst G.J., Peelen L.M., van Klei W.A., Buhre W.F., Kalkman C.J., van Wolfswinkel L. (2013). Myocardial injury after noncardiac surgery and its association with short-term mortality. Circulation.

[32] Taschner A., Kabon B., Graf A., Adamowitsch N., von Sonnenburg M.F., Fraunschiel M., Horvath K., Fleischmann E., Reiterer C. (2022). Perioperative Supplemental Oxygen and Postoperative Copeptin Concentrations in Cardiac-Risk Patients Undergoing Major Abdominal Surgery—A Secondary Analysis of a Randomized Clinical Trial. J. Clin. Med..

